# Preterm meconium‐stained amniotic fluid: A red flag for infection and adverse perinatal outcomes

**DOI:** 10.1002/ijgo.70337

**Published:** 2025-06-26

**Authors:** Raneen Abu Shqara, Neta Hoffman, Lior Lowenstein, Maya Frank Wolf

**Affiliations:** ^1^ Raya Strauss Wing of Obstetrics and Gynecology Galilee Medical Center Nahariya Israel; ^2^ Azrieli Faculty of Medicine Bar Ilan University Safed Israel

**Keywords:** antibiotic prophylaxis, chorioamnionitis, GBS, maternal infections, meconium‐stained amniotic fluid, neonatal complications, preterm birth, respiratory distress syndrome

## Abstract

**Objective:**

Meconium‐stained amniotic fluid (MSAF) is uncommon in preterm deliveries and raises concerns about maternal and neonatal infectious risks. We aimed to evaluate maternal, perinatal, and microbiological outcomes in preterm deliveries complicated by MSAF compared to clear amniotic fluid (AF) and to assess outcomes in preterm versus term deliveries with MSAF.

**Methods:**

This retrospective cohort study included singleton pregnancies delivered at 24–42 weeks at a tertiary hospital (March 2020–May 2024), with documented AF color. Chorioamniotic swab culture results were analyzed, and clinical outcomes compared using univariate and multivariate analyses.

**Results:**

Of 1550 preterm deliveries, 93 (6%) were complicated by MSAF. For preterm births with MSAF compared to AF, the rates were higher of clinical chorioamnionitis, puerperal endometritis, maternal bacteremia, and adverse neonatal outcomes, including respiratory distress syndrome and the need for ventilation (*P* < 0.001 for all). Positive chorioamniotic swab cultures were more frequent in preterm MSAF than preterm clear AF (26 [28.0%] vs. 168 [11.5%], *P* < 0.001), with higher isolation of Enterobacteriaceae (*P* = 0.030) and group B streptococcus (GBS; *P* = 0.040). Comparing preterm versus term deliveries, among those complicated by MSAF, the rates were higher of clinical chorioamnionitis, endometritis, maternal bacteremia, early‐onset sepsis, and positive chorioamniotic cultures (26 [28.0%] vs. 220 [10.0%], *P* < 0.001), particularly Enterobacteriaceae (*P* = 0.001) and *Enterococcus* spp. (*P* = 0.003).

**Conclusion:**

Preterm deliveries with MSAF, compared to preterm clear AF and term MSAF, were associated with increased maternal infections, neonatal morbidity, and distinct microbial colonization. Chorioamniotic swab cultures might aid in identifying high‐risk preterm births requiring targeted infection management.

## 1 INTRODUCTION

Meconium‐stained amniotic fluid (MSAF), caused by the intrauterine passage of meconium into the amniotic fluid (AF), affects 8%–20% of term pregnancies and only 2%–5% of pregnancies of less than 37 weeks.^1^, ^2^ The maturation of the fetal gastrointestinal system, specifically the complete innervation of the anal sphincter by the 34th week, likely contributes to meconium passage in term pregnancies.^1^, ^3^ However, the mechanisms driving meconium passage in preterm pregnancies remain poorly understood. MSAF has been suggested as a marker for intrauterine microbial invasion.^4^, ^5^ Among 707 patients with preterm labor and intact membranes, MSAF was identified in 4.2%.^5^ The greater frequency among those with MSAF than with clear AF (33% vs. 11%) indicates a potential association between MSAF and microbial invasion.

Although MSAF is common, only approximately 5% of the exposed neonates develop meconium aspiration syndrome (MAS).^6^ MSAF has been associated with hypoxic–ischemic encephalopathy,^7^ neonatal sepsis,^8^ neonatal seizures,^9^ and cerebral palsy.^10^ Despite its association with hypoxic–ischemic encephalopathy,^8^ most neonates born to mothers with MSAF do not exhibit evidence of metabolic acidemia.^11^, ^12^

It remains unclear whether the implications of MSAF differ between preterm and term births. Among 172 women with preterm labor, among those with MSAF compared to clear AF, the incidence was higher of clinical chorioamnionitis (15.0% vs. 4.3%). However, neonatal complications were similar between the groups.^13^ We aimed to compare perinatal and infectious outcomes in preterm deliveries, between those with MSAF and those with clear AF. Additionally, we examined differences in perinatal and microbiological outcomes between preterm and term deliveries complicated by MSAF.

## 2 MATERIALS AND METHODS

### 2.1 Study population

This retrospective study was conducted on pregnant women admitted to a tertiary hospital between March 2020 and May 2024. The study included singleton pregnancies with gestational ages between 24 and 42 weeks, in which AF color was documented during labor (classified as either clear AF or MSAF). The study included two comparisons. The first compared women with spontaneous preterm delivery (<36 6/7 weeks) between those complicated by MSAF and those with clear AF. The second compared deliveries complicated by MSAF between those that were preterm and term. The subgroup that delivered at term, complicated by MSAF, was derived from the same institutional population during the study period. Exclusion criteria included multiple gestations, major fetal anomalies, intrauterine fetal death, and elective cesarean delivery. Institutional review board approval was obtained, and informed consent was waived due to the retrospective design.

### 2.2 Outcomes of interest

Primary infectious outcomes of interest included clinical chorioamnionitis and neonatal early onset sepsis (EOS). Secondary maternal outcomes included intrapartum fever, puerperal endometritis, cesarean delivery, and postpartum hemorrhage. Secondary neonatal outcomes included 5‐min Apgar score <7, umbilical cord pH <7.2, neonatal intensive care unit (NICU) admission, respiratory distress syndrome (RDS), and the need for invasive ventilation support. These were all components of the composite adverse perinatal outcome.

Meconium‐stained amniotic fluid was categorized as thin, intermediate, or thick according to the clinician's documentation at the time of delivery. The rate of thick meconium was compared between term and preterm deliveries. Microbiological analysis was conducted on post‐delivery chorioamniotic swab cultures obtained routinely from the placental membranes, with specific focus GBS, Enterobacteriaceae, anaerobes, and *Enterococcus* spp. Chorioamnionitis was defined as maternal temperature ≥39.0°C or maternal temperature of 38.0–38.9°C accompanied by one additional clinical risk factor, such as maternal leukocytosis (>15 000/mm^3^), purulent cervical drainage, or fetal tachycardia (>160 beats/min).^14^ Clinical chorioamnionitis was treated by ampicillin (2 g four times daily) and gentamicin (240 mg once daily).^15^, ^16^ Puerperal endometritis was diagnosed according to body temperature ≥38°C, in the absence of any other cause, along with an associated clinical finding such as uterine tenderness, purulent lochia, tachycardia, or abdominal pain. Based on the Kaiser Permanente electronic calculator,^17^ culture‐confirmed EOS was identified by isolating a pathogenic organism from a blood culture, accompanied by additional clinical or laboratory evidence of infection occurring within 7 days post‐birth.

### 2.3 Statistical analysis

Continuous variables are presented as means with standard deviations, or medians with ranges, while categorical variables are expressed as frequencies and percentages. We used Student's *t*‐test to compare normally distributed continuous variables and the Mann–Whitney *U*‐test for non‐normally distributed data. For categorical variables, comparisons were made using the *χ* ^2^‐test or Fisher's exact test when cell counts were low. Relative risk (RR) with 95% confidence intervals (CI) was calculated to estimate the likelihood of infectious and adverse perinatal outcomes associated with MSAF in the preterm versus term births. A multivariate logistic regression model aimed to predict clinical chorioamnionitis controlled for maternal age, delivery number, delivery week, urinary tract infection in pregnancy, GBS colonization, rupture of membranes (ROM) duration, and MSAF. A multivariate logistic regression model aimed to predict composite perinatal outcome controlled for maternal age, delivery number, delivery week, GBS colonization, ROM duration, and MSAF. All the analyses were conducted using IBM SPSS Statistics for Windows, version 27.0 (IBM Corp., Armonk, NY, USA), with a significance level set at *P* < 0.05.

## 3 RESULTS

### 3.1 Study population

**FIGURE 1:**
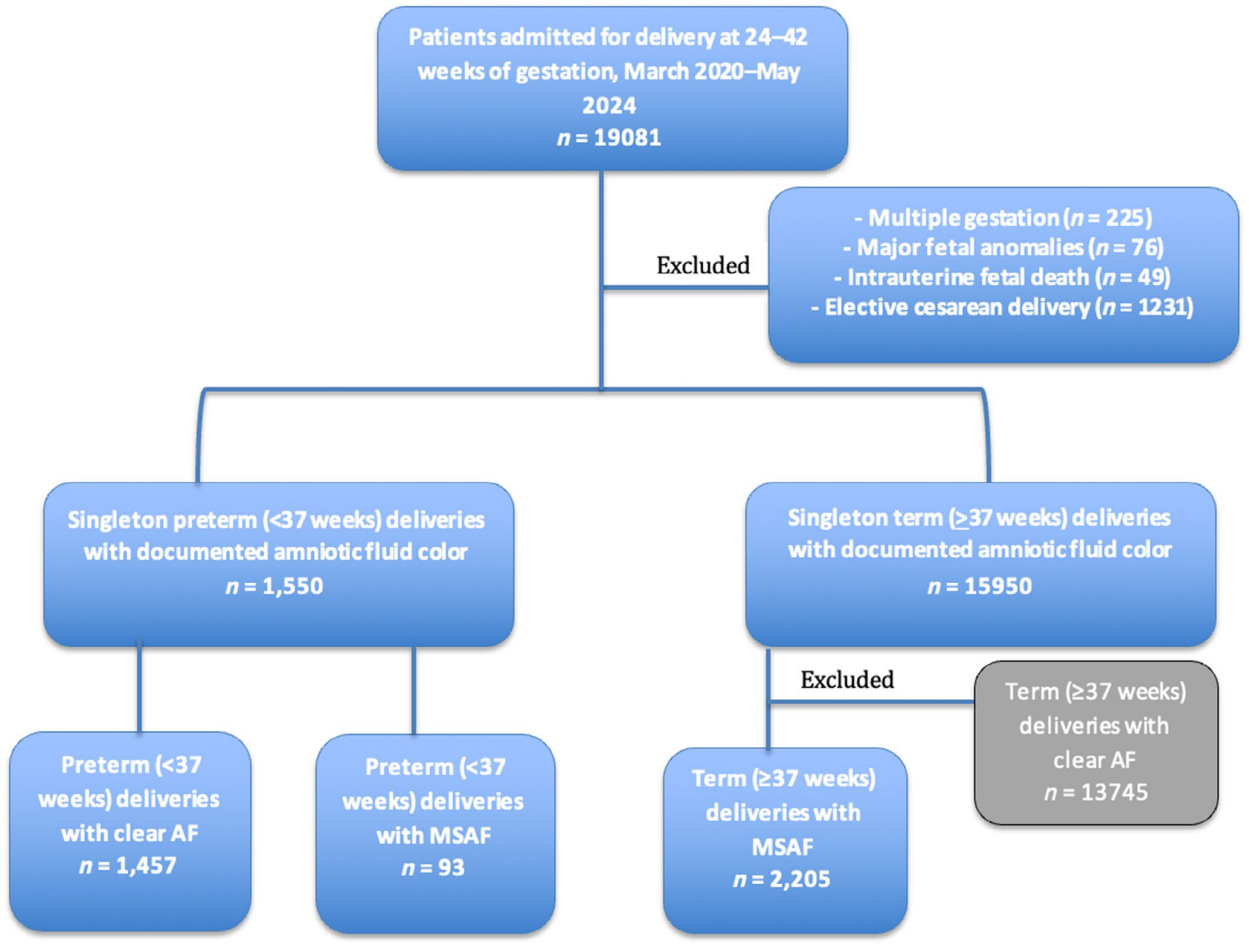
Flow diagram of patient selection. AF, amniotic fluid; MSAF, meconium‐stained amniotic fluid.

A total of 1550 women with preterm labor were included in the study; 93 (6%) presented with MSAF during labor and 1457 with clear AF (Figure 1). Among those with MSAF, meconium was classified as thick in one (1.1%), thin in 22 (23.7%), and intermediate in the remaining 70 (75.3%). Baseline characteristics, including maternal age, pregnancy number, and delivery number, were similar between the groups (Table 1). Among preterm deliveries, for those complicated by MSAF compared to those with clear AF, the median gestational age was higher (36.1 [24.4–36.6] vs. 35.4 [24–36.6] weeks; *P* < 0.001). Among women with MSAF compared to clear AF, the rate of GBS colonization was higher (18.3% vs. 10.4%, *P* = 0.025). The median duration of ROM did not differ significantly between the groups (7.2 [1.7–1520.1] vs. 10.7 [1.3–1791.6] hours; *P* = 0.347).

**TABLE 1:** Patients' characteristics and obstetrical history data according to presence of MSAF in preterm births.

	Clear AF *N* = 1457	MSAF *N* = 93	*P*‐value
Maternal age	30.6 (18.7–45.6)	30.7 (18.6–47.2)	0.927
Pregnancy number	2 (1–14)	3 (1–9)	0.378
Delivery number	2 (1–6)	2 (1–9)	0.505
Diabetes mellitus	188 (12.9)	10 (10.8)	0.633
Hypertension	89 (6.1)	2 (2.1)	0.167
Previous cesarean delivery	90 (6.2)	3 (3.2)	0.733
Pregnancy week	35.4 (24–36.6)	36.1 (24.4–36.6)	<0.001
GBS colonization	152 (10.4)	17 (18.3)	0.025
Urinary tract infection	126 (8.7)	4 (4.3)	0.177
Premature rupture of membranes	396 (27.2)	22 (23.7)	0.547
ROM duration (hours)	10.7 (1.3–1791.6)	7.2 (1.7–1520.1)	0.347

*Note*: Data are presented in *N* (%), median (range), or mean (standard deviation).

Abbreviations: AF, amniotic fluid; GBS, group B streptococcus; MSAF, meconium‐stained amniotic fluid; ROM, rupture of membranes.

### 3.2 Outcomes in preterm birth stratified by meconium‐stained amniotic fluid versus clear amniotic fluid

#### Maternal outcomes

**FIGURE 2:**
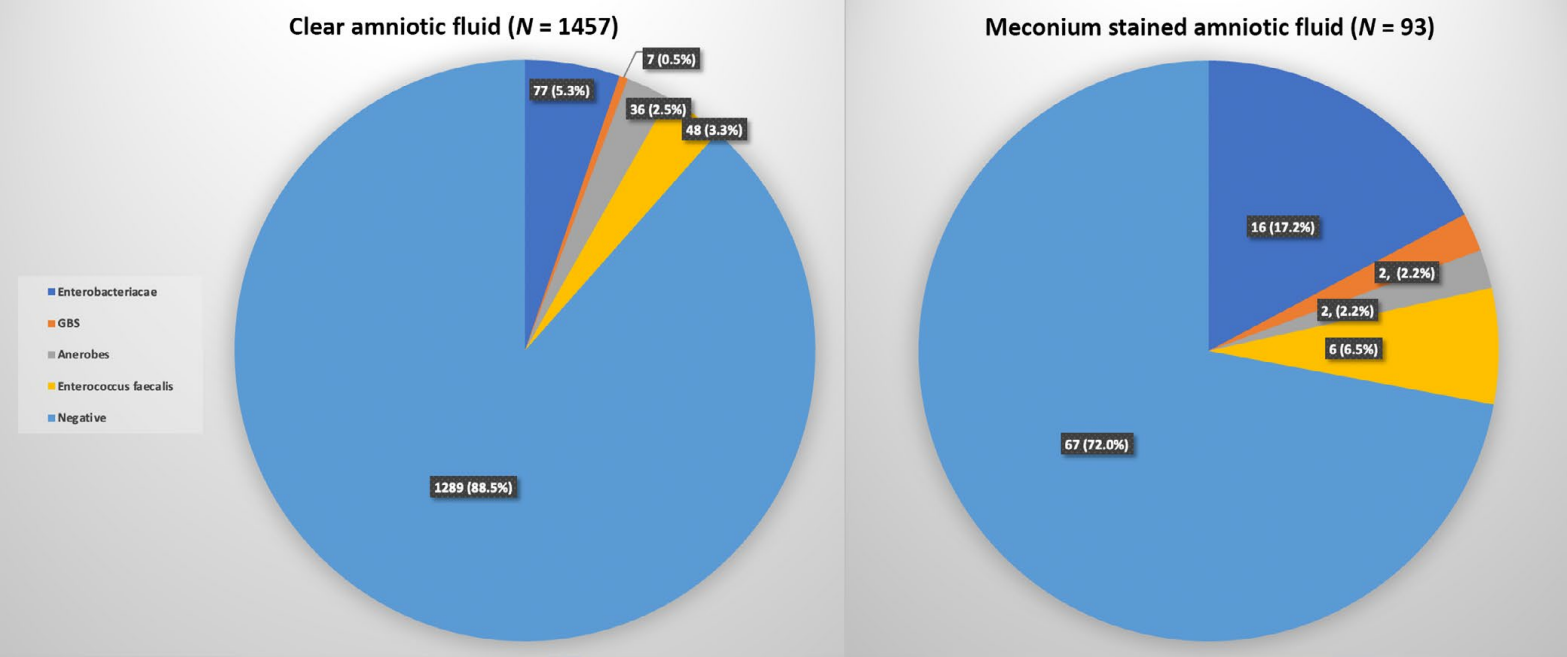
Chorioamniotic swab culture results in preterm births: A comparison between MSAF and clear AF cases. AF, amniotic fluid; GBS, group B streptococcus; MSAF, meconium‐stained amniotic fluid.

Maternal outcomes are detailed in Table 2. Among the preterm deliveries complicated by MSAF, compared to clear AF, the rates were higher rates of intrapartum fever (16 [17.2%] vs. 22 [1.5%], *P* = 0.019), clinical chorioamnionitis (7 [7.5]% vs. 9 [0.6%], *P* < 0.001), puerperal endometritis (4.3% vs. 0.1%, *P* < 0.001), maternal bacteremia (2 [2.2]% vs. 1 [0.06]%, *P* < 0.001), and cesarean delivery due to fetal distress (14 [15.1%] vs. 129 [8.9%], *P* = 0.045). The rates were similar for overall cesarean delivery, manual placenta removal, and postpartum hemorrhage. Neonatal outcomes are shown in Table 2. Among the preterm births, for the neonates born to mothers with MSAF, compared to clear AF, the rates were higher of 5‐min Apgar score <7 (20 [21.5%] vs. 4 [0.3%], *P* = 0.027), umbilical cord pH levels <7.2 (9 [9.7%] vs. 63 [4.3%], *P* = 0.035), RDS (19 [20.4%] vs. 170 [11.7%], *P* < 0.001), and the need for invasive ventilation (18 [19.4%] vs. 66 [4.5%], *P* < 0.001). NICU admission rates were similar. Microbiological findings are shown in Figure 2. In preterm births, positive chorioamniotic swab cultures were more prevalent among those with MSAF, compared to clear AF (26 [28.0%] vs. 168 [11.5%], *P* < 0.001). Specifically, Enterobacteriaceae (16 [17.2%] vs. 77 [5.3%], *P* = 0.030) and GBS (2 [2.2%] vs. 7 [0.5%], *P* = 0.040) were isolated more frequently in preterm births complicated by MSAF. Anaerobes and *Enterococcus faecalis* rates were similar between the groups.

**TABLE 2:** Maternal and neonatal outcomes according to presence of MSAF in preterm births.

	Clear AF *N* = 1457	MSAF *N* = 93	*P*‐value
Maternal outcomes			
Intrapartum fever	22 (1.5)	16 (17.2)	0.019
Clinical chorioamnionitis	9 (0.6)	7 (7.5)	<0.001
Cesarean delivery	765 (52.5)	41 (44.1)	0.134
Cesarean delivery due to fetal distress	129 (8.9)	14 (15.1)	0.045
Manual removal of placenta	37 (2.5)	2 (2.2)	>0.99
Postpartum hemorrhage	76 (5.2)	3 (3.2)	0.133
Postpartum fever	57 (3.1)	7 (7.5)	<0.001
Puerperal endometritis	2 (0.1)	4 (4.3)	<0.001
Maternal bacteremia	1 (0.06)	2 (2.2)	<0.001
Neonatal outcomes			
Apgar‐5 <7	4 (0.3)	20 (21.5)	0.027
pH <7.2	63 (4.3)	9 (9.7)	0.035
NICU admission	787 (54.0)	43 (46.2)	0.069
Respiratory distress syndrome	170 (11.7)	19 (20.4)	<0.001
Invasive ventilation	66 (4.5)	18 (19.4)	<0.001
Noninvasive ventilation	244 (16.7)	10 (10.8)	0.117
Meconium aspiration syndrome	0 (0)	0 (0)	>0.99
Asphyxia	2 (0.1)	0 (0)	>0.99
NEC	15 (1.0)	1 (1.1)	>0.99
IVH	67 (4.6)	3 (3.2)	0.795
EOS	5 (0.3)	1 (1.1)	0.311

Abbreviations: AF, amniotic fluid; EOS, early onset sepsis; IVH, intraventricular hemorrhage; MSAF, meconium‐stained amniotic fluid; NEC, necrotizing enterocolitis; NICU, neonatal intensive care unit.

### 3.3 Outcomes of deliveries complicated by MSAF according to preterm versus term onset of labor

Maternal outcomes in preterm (*n* = 93) versus term deliveries (*n* = 2205) complicated by MSAF are presented in Table 3. Among the term deliveries complicated by MSAF (*n* = 2205), meconium was classified as thick in 218 (9.9%), intermediate in 1426 (64.7%), and thin in 561 (25.4%). The rate of thick meconium was lower in preterm than term deliveries (1 [1.1%] vs. 218 [9.9%]; RR 0.11, 95% CI: 0.02–0.77; *P* = 0.002). In preterm compared to term deliveries complicated by MSAF, the risks of clinical chorioamnionitis (RR 4.21; 95% CI, 1.92–9.13; *P* = 0.002), puerperal endometritis (RR 7.51; 95% CI, 2.55–23.12; *P* = 0.003), and maternal bacteremia (RR 9.12; 95% CI, 1.81–45.35; *P* = 0.007) were significantly higher. In preterm compared to term deliveries, the risks of EOS (RR 11.43; 95% CI, 1.11–124.32; *P* = 0.046 ), 5‐min Apgar score <7 (RR 90.91; 95% CI, 34.82–237.21; *P* < 0.001), and umbilical cord pH <7.2 (RR 2.16; 95% CI, 1.15–4.11; *P* = 0.024) were significantly higher. Among deliveries complicated by MSAF, significant differences were not observed in the rates of MAS between those that were preterm and term. The RR of positive chorioamniotic swab cultures was significantly higher in the preterm group (2.71; 95% CI, 1.96–3.81; *P* < 0.001). The RR of Enterobacteriaceae and *Enterococcus* spp. isolated from chorioamniotic swab cultures were higher in preterm than term deliveries (2.22; 95% CI, 1.41–3.55; *P* = 0.001, and 7.51; 95% CI, 3.11–18.76; *P* = 0.003, respectively).

**TABLE 3:** Relative risk of infectious outcomes in preterm versus term deliveries complicated by MSAF.

	Term *N* = 2205	Preterm *N* = 93	RR (95% CI)	*P*‐value
Meconium thickness				
Thick	218 (9.9)	1 (1.1)	0.11 (0.02–0.77)	0.002
Maternal outcomes				
Clinical chorioamnionitis	38 (1.7)	7 (7.5)	4.21 (1.92–9.13)	0.002
Puerperal endometritis	12 (0.5)	4 (4.3)	7.51 (2.55–23.12)	0.003
Maternal bacteremia	5 (0.2)	2 (2.2)	9.12 (1.81–45.35)	0.007
Neonatal outcomes				
Apgar‐5 <7	5 (0.2)	20 (21.5)	90.91 (34.82–237.21)	<0.001
pH <7.2	97 (4.4)	9 (9.7)	2.16 (1.15–4.11)	0.024
MAS	24 (1.1)	0 (0)	0.51 (0.03–7.81)	0.605
EOS	2 (0.1)	1 (1.1)	11.43 (1.11–124.32)	0.046
Microbiological outcomes				
Positive	220 (10.0)	26 (28.0)	2.71 (1.96–3.81)	<0.001
Enterobacteriaceae spp.	168 (7.6)	16 (17.2)	2.22 (1.41–3.55)	0.001
Anaerobes	18 (0.8)	2 (2.2)	2.56 (0.64–10.74)	0.205
GBS	16 (0.7)	2 (2.2)	2.85 (0.74–12.11)	0.174
*Enterococcus*	18 (0.8)	6 (6.5)	7.51 (3.11–18.76)	0.003

Abbreviations: EOS, early onset sepsis; GBS, group B streptococcus; MAS, meconium aspiration syndrome; MSAF, meconium‐stained amniotic fluid.

### 3.4 Multivariate analysis

Table 4 presents the results of the multivariate logistic regression model for predicting clinical chorioamnionitis. The presence of MSAF (odds ratio [OR] 12.31, 95% CI 4.20–35.62, *P* < 0.001) and lower gestational age at delivery (OR 0.90, 95% CI 0.70–0.91, *P* = 0.018) were independently associated with clinical chorioamnionitis. Table 5 presents the results of the multivariate logistic regression model for predicting composite perinatal adverse outcomes. The presence of MSAF (OR 6.07, 95% CI 2.25–18.41, *P* < 0.001) and lower gestational age at delivery (OR 0.7, 95% CI 0.20–0.91, *P* = 0.024) were independently associated with an increased risk of composite adverse perinatal outcome.

**TABLE 4:** Multivariate analysis for prediction of clinical chorioamnionitis.

Variable	Odds ratio	95% CI	*P*‐value
Maternal age	0.91	0.72–1.01	0.054
Delivery number	1.43	0.90–2.11	0.099
Delivery week	0.90	0.70–0.91	0.018
Urinary tract infection in pregnancy	0.12	0.11–1.10	0.998
GBS colonization	1.33	0.31–4.80	0.742
ROM duration	1.01	0.96–1.12	0.354
MSAF	12.31	4.20–35.62	<0.001

Abbreviations: CI, confidence interval; GBS, group B streptococcus; MSAF, meconium‐stained amniotic fluid; ROM, rupture of membranes.

**TABLE 5:** Multivariate analysis for prediction of adverse perinatal outcomes.

Variable	Odds ratio	95% CI	*P*‐value
Maternal age	1.23	0.71–1.50	0.145
Delivery number	1.91	0.61–2.62	0.245
Delivery week	0.70	0.21–0.90	0.024
GBS colonization	1.70	0.41–6.09	0.543
ROM duration	1.16	0.90–1.55	0.367
MSAF	6.07	2.25–18.41	<0.001

Abbreviations: CI, confidence interval; GBS, group B streptococcus; MSAF, meconium‐stained amniotic fluid; ROM, rupture of membranes.

## 4 DISCUSSION

### 4.1 Principal findings

Among preterm deliveries, for those with MSAF compared to AF, the rates were higher of intrapartum fever, clinical chorioamnionitis, puerperal endometritis, maternal bacteremia, and cesarean delivery for fetal distress. Neonatal outcomes were also worse for those with MSAF, as evident by higher rates of low 5‐min Apgar scores, umbilical cord pH <7.2, RDS, and the need for invasive ventilation. The EOS rates were similar between the groups. When comparing MSAF cases between preterm and term deliveries, the preterm group had higher rates of clinical chorioamnionitis, puerperal endometritis, maternal bacteremia, cesarean delivery for fetal distress, and EOS.

### 4.2 Interpretation

Among the women with preterm births, for those with MSAF compared with clear AF, the rates were higher of clinical chorioamnionitis, puerperal endometritis, and positive chorioamniotic swab. Among the women with preterm births, we also observed increased rates of maternal bacteremia for those with MSAF compared to AF. These findings are consistent with a 6‐year study involving 86 590 deliveries, which reported a 0.84% incidence of peripartum bacteremia in early preterm births. There, MSAF was more prevalent in the bacteremia than the non‐bacteremia group (22.7% vs. 6.9%).^18^ However, the microbiological outcomes of MSAF in preterm births were not detailed in that study. Two mechanisms have been proposed to explain the correlation between MSAF and peripartum infections. The first suggests that fetal meconium passage may occur after swallowed bacteria stimulate bowel peristalsis.^19^ The second posits that MSAF increases the risk of microbial invasion. This is supported by experimental evidence that the meconium reduces antimicrobial activity of AF.^4^

In preterm deliveries, we found higher rates of Enterobacteriaceae and GBS‐positive chorioamniotic swab cultures in those with MSAF compared to clear AF. Previously, even minimal concentrations of meconium were shown to significantly accelerated GBS growth; the growth rates of GBS and *Escherichia coli* becoming comparable only at higher meconium concentrations.^20^

Perinatal complications were higher for those with MSAF versus clear AF. Previously, the correlation was evident at a more advanced gestational age.^21^ We report higher rates of RDS (20.4%) and the need for invasive ventilation (19.4%) in women with preterm birth complicated by MSAF. Our rates are comparable to a report of similar rates of RDS associated with MSAF, in which most presentations were mild to moderate and only 21.7% severe.^22^ None of our preterm births were complicated by MAS. This aligns with the reporting of a MAS rate of only 0.067% in preterm births.^23^ However, our sample size might have been insufficient to detect such a rare occurrence. Additionally, while a correlation between MSAF was previously demonstrated in preterm births, together with an increased risk of intraventricular hemorrhage,^24^ we did not observe such a correlation. Although we did not assess long‐term outcomes, MSAF in preterm births has been linked to a higher risk of cerebral palsy.^25^

MSAF generally occurs at term.^26^ However, higher rates of clinical chorioamnionitis, puerperal endometritis, and maternal bacteremia have been observed in preterm births with MSAF than term births with MSAF. Intrauterine infection was implicated in at least 40% of preterm births.^27^ Notably, the correlation between MSAF and clinical chorioamnionitis remained statistically significant even after adjusting for potential confounders, including delivery week. Among those with MSAF, for preterm compared to term births, perinatal outcomes were worse, and the rates were higher of low Apgar scores (<7 at 5 min) and reduced umbilical cord pH. However, notably, Apgar scores typically improve with increasing gestational age, as reported in previous studies.^28^, ^29^

Obstetrical conditions such as post‐term delivery, oligohydramnios, uteroplacental insufficiency, and impaired meconium clearance have been implicated in the development of thick MSAF.^6^, ^11^ This was more frequently observed in term than preterm deliveries in our cohort.

No significant difference was observed in the rate of MAS between preterm and term deliveries, despite MAS being reported in approximately 5% of those with MSAF and typically described as a severe complication of term newborns.^6^ We report MAS in 1.1% of term deliveries, yet the difference between term and preterm deliveries was not statistically significant. A larger sample size might be required to detect a meaningful difference in MAS rates.

### 4.3 Clinical implications

Based on our findings, we propose that in term pregnancies, MSAF is likely more associated with gut maturity, whereas in preterm pregnancies, it appears to be more closely linked to infections. Hence, we recommend that infection should be carefully ruled out in preterm births complicated by MSAF, particularly given the observed correlation between MSAF and clinical chorioamnionitis, as well as EOS.^6^ The higher rates of positive chorioamniotic swab cultures in preterm deliveries, especially for Enterobacteriaceae and *Enterococcus* spp., highlight the influence of gestational age on the microbial composition of meconium. Previous research has also shown that the presence of Enterobacteriaceae and *Enterococcus* spp. in meconium is inversely correlated with gestational age and can trigger inflammatory responses, suggesting a potential causative role in preterm birth.^11^ These together reinforce the need for targeted microbiological assessment in preterm deliveries complicated by MSAF and tailored antibiotic management strategies.

### 4.4 Strengths and limitations

Our study's novelty lies in its comprehensive comparison of preterm deliveries with and without MSAF and on the direct comparison between preterm and term births with MSAF. However, this study has several limitations. These include its retrospective design, its being underpowered to detect significant differences in MAS rates between preterm and term deliveries, and the lack of detailed data on long‐term neonatal outcomes.

## 5 CONCLUSION

Preterm MSAF compared to clear AF was associated with an increased risk of maternal infections, neonatal complications, and specific microbial colonization patterns. These findings emphasize the importance of exploring targeted chorioamniotic swab cultures in preterm births associated with MSAF and the potential role of antibiotic prophylaxis in reducing adverse outcomes.

## AUTHOR CONTRIBUTIONS

R.A.S., N.H., and L.L. were involved in conceiving, planning, carrying out, analyzing and writing the work. M.F.W. was involved in conceiving, analyzing, and writing and revising the work.

## FUNDING INFORMATION

The authors declare that no funds, grants, or other support were received during the preparation of this manuscript.

## CONFLICT OF INTEREST STATEMENT

The authors have no relevant financial or non‐financial interests to disclose.

## Data Availability

Reasonable requests for data that do not identify the patient will be shared by the corresponding author.
